# Chronic Kidney Diseases and Acute Kidney Injury in Patients With COVID-19: Evidence From a Meta-Analysis

**DOI:** 10.3389/fmed.2020.588301

**Published:** 2020-11-03

**Authors:** Yangzhong Zhou, Qidong Ren, Gang Chen, Qiao Jin, Quexuan Cui, Huiting Luo, Ke Zheng, Yan Qin, Xuemei Li

**Affiliations:** ^1^Department of Nephrology, Peking Union Medical College, Peking Union Medical College Hospital, Chinese Academy of Medical Sciences, Beijing, China; ^2^School of Medicine, Tsinghua University, Beijing, China

**Keywords:** COVID-19, chronic kidney disease, acute kidney injury, severity, prognosis

## Abstract

Renal involvement has been implicated in coronavirus disease 2019 (COVID-19), but the related prevalence and prognosis were largely unknown. In this meta-analysis, we searched the literature from PubMed, Embase, through bioRxiv, and medRxiv until April 26, 2020. Studies reporting chronic kidney diseases (CKDs) and/or acute kidney injury (AKI) were included. Demographics, relevant data of disease severity, and patient's prognosis were extracted and aggregated. Twenty-one thousand one hundred sixty-four patients from 52 peer-reviewed studies were included. Thirty-seven studies (*n* = 16,922) reported CKD in COVID-19 patients at diagnosis, and the pooled prevalence was 3.52% (95% CI, 1.98–5.48%; *I*^2^ = 93%). Subgroup analysis showed that CKD prevalence was higher in severe cases [odds ratio (OR), 3.42; 95% CI 2.05–5.61; *I*^2^ = 0%] compared to those with non-severe disease and deceased cases (6.46, 3.40–12.29; *I*^2^ = 1%) compared with survivors. Pooled prevalence of CKD was lower in Chinese patients (2.56%; 95% CI, 1.79–3.47%; *I*^2^ = 80%) compared to those outside of China (6.32%; 95% CI, 0.9–16.12%; *I*^2^ = 93%) (*p* = 0.08). The summary estimates for AKI prevalence was 11.46% (95% CI, 6.93–16.94%). Patients with AKI had a higher prevalence of developing into severe cases (OR, 6.97; 95% CI, 3.53–13.75; *I*^2^ = 0%) and mortality risk (45.79, 36.88–56.85; *I*^2^ = 17%). The prevalence estimates of CKD or AKI were not significantly different from preprint publications (*p* > 0.05). Our study indicates that renal condition, either in CKD or AKI, is associated with COVID-19 prognosis, and taking care of such patients needs further awareness and investigations.

## Introduction

Since December 2019, the coronavirus disease 2019 (COVID-19) has rapidly developed into a global pandemic (1), with more than four million cases confirmed until the beginning of May 2020 (2). The variable clinical course of COVID-19 ranged from asymptomatic infection to severe cases, and 5–6% may need intensive care unit (ICU) admission (3–5).

The kidney is not a bystander during the disease course. Of the hospitalized patients, 43.9–75.4% had evidence of abnormal kidney function, and 5.1–10.5% of them presented acute kidney injury (AKI) with an excess mortality rate (6, 7). Postmortem evaluations detected viral antigen in tubular epithelial cells and revealed severe acute tubular injury (8). Moreover, preexisting chronic kidney disease (CKD) could reasonably act as an ominous clinical predictor and associate with high mortality (6). Accumulating evidence has suggested that patients with chronic comorbidities were more likely to develop into severe cases (9). Data from multiple cohorts and meta-analyses have listed aging, hypertension, diabetes, and cardiovascular disease as adverse prognostic markers for COVID-19 patients (10, 11), which were also commonly seen in CKD (12, 13).

While AKI is often carefully monitored in such a complicated infectious disease (14), CKD, affecting over 10% of the general population, is often neglected (15). Data on the risk of patients with CKD are limited and highly variable, especially in a scenario of lung-dominated infectious disease (16). Still, it is difficult to picture the relationship between kidney diseases and the prognosis of COVID-19 infection in the surgent literature.

Therefore, we conducted a systematic review and meta-analysis of studies on the clinical characteristics of COVID-19 patients, irrespective of intervention or comparator. We focused on the prevalence of CKD and AKI, as well as their association with poor prognosis.

## Results

### Search Results and Characteristics of Included Studies

**Figure 1:**
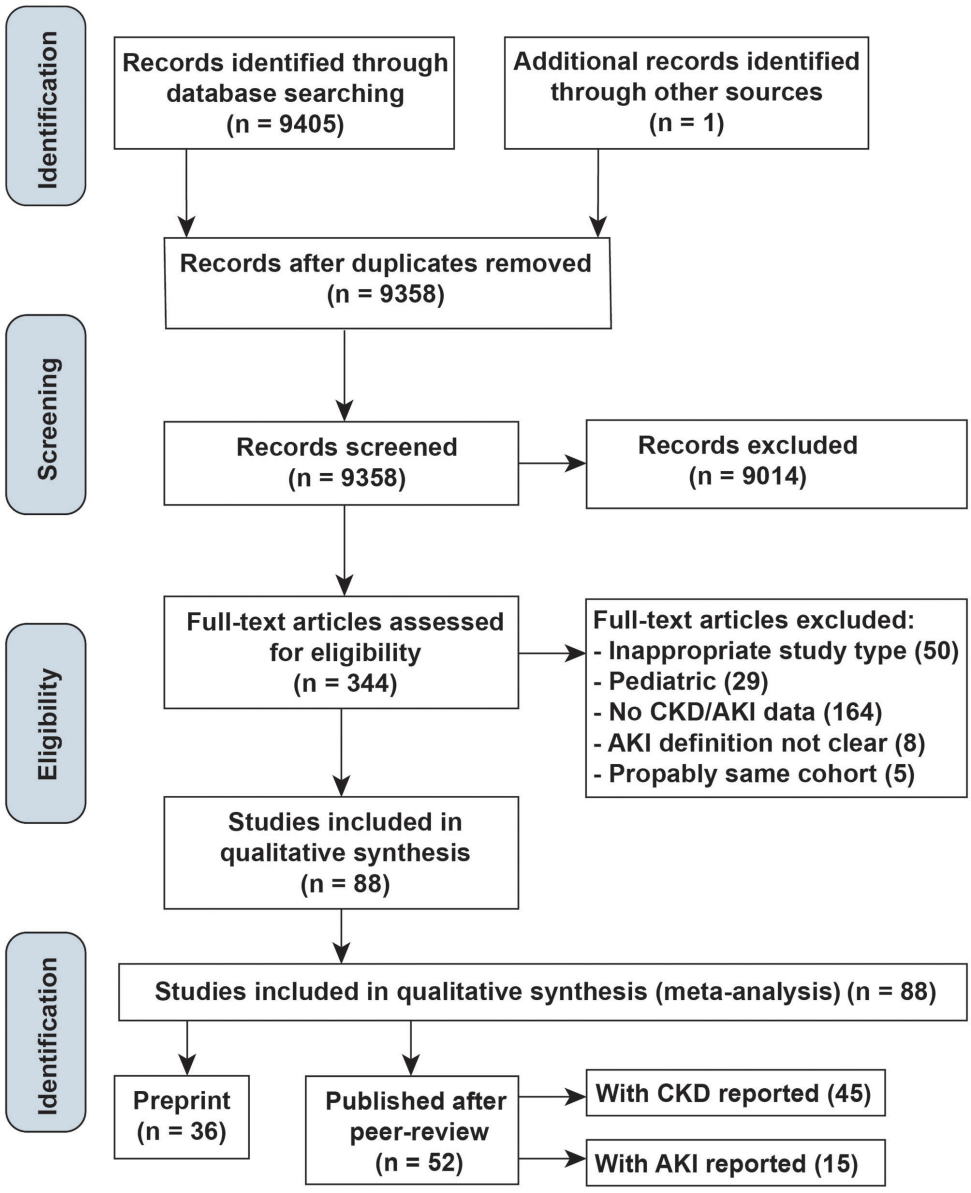
Study selection diagram.

The literature search yielded 9,405 articles and one additional record (6), of which 344 were reviewed in full text (Figure 1). Of these, 52 peer-reviewed articles (45 reported CKD, 15 reported AKI) and 36 preprint manuscripts met the inclusion criteria. Most studies identified by our search, but excluded from the final review, were not in proper study types, not relevant populations, or did not report CKD/AKI events, lacked AKI definitions, or were duplicates of cohorts already identified.

The characteristics of the included studies are summarized in Supplementary Table 2. All the included studies were cohort studies. Their sample sizes ranged from 5 to 5,700 participants, and follow-up durations were reported in 22 studies, ranging from 5 days to 1 month. Of all, 33 were conducted in a single center (5–7, 9, 17–45), while 19 studies were reporting data from multiple centers (4, 46–63). Of the 52 studies, 40 were from China; especially, 28 of them include data from the designated hospitals in Wuhan, and 12 were from other countries, including the US, Italy, France, Korea, and Singapore.

The median age of study participants ranged from 41 to 72 years, and 32.93–73.17% of them were male. Thirty-eight studies recruited consecutive patients in their centers, while the rest specified their participants as deceased, ICU patients, elderly, medical workers, or others.

Quality assessment of studies was performed according to the specific analysis they were applied separately, and the Newcastle–Ottawa Scale (NOS) scores of all the articles included were no <6.

### Prevalence of Chronic Kidney Disease and Associations With Prognosis

**Figure 2:**
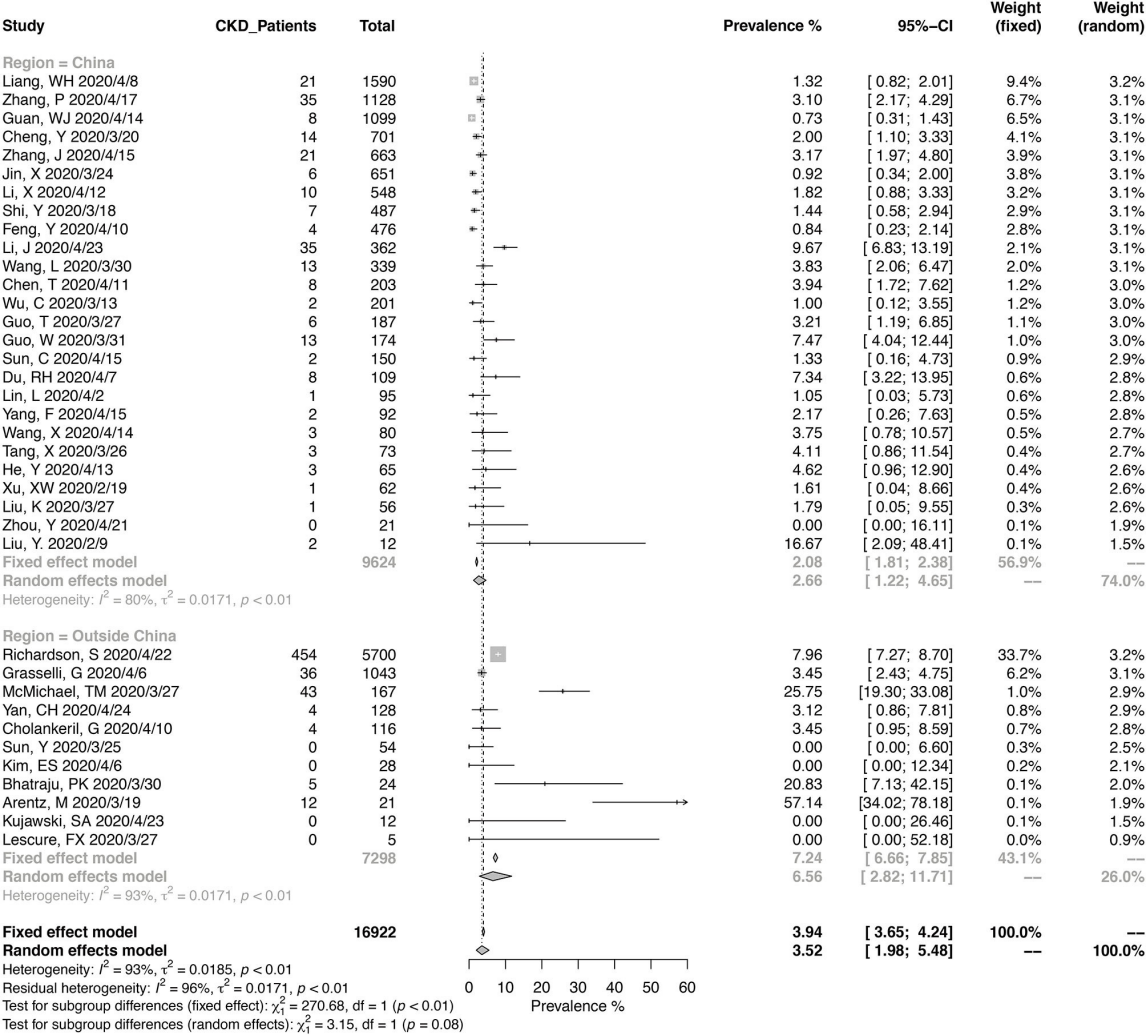
Pooled prevalence of chronic kidney disease in coronavirus disease 2019 (COVID-19) patients.

A total of 16,922 COVID-19 patients from 37 studies were included in the meta-analysis for the prevalence of CKD (Figure 2). The pooled prevalence of CKD in these patients was 3.52% (95% CI, 1.98–5.48%), and the heterogeneity of the studies was significant (*I*^2^ = 93%, *p* < 0.01). Twenty-six studies were from China, and 11 were from other countries. Of the Chinese studies, 16 studies were reporting data from Wuhan in Hubei province. The pooled CKD prevalence was higher in the studies outside of China (6.56%; 95% CI, 2.82–11.71%) compared with those from China (2.66%; 95% CI, 1.22–4.65%), with a borderline level of statistical significance (*p* = 0.08). The heterogeneity was significant in either Chinese studies (*I*^2^ = 80%, *p* < 0.01) and the others (*I*^2^ = 93%, *p* < 0.01).

**Figure 3:**
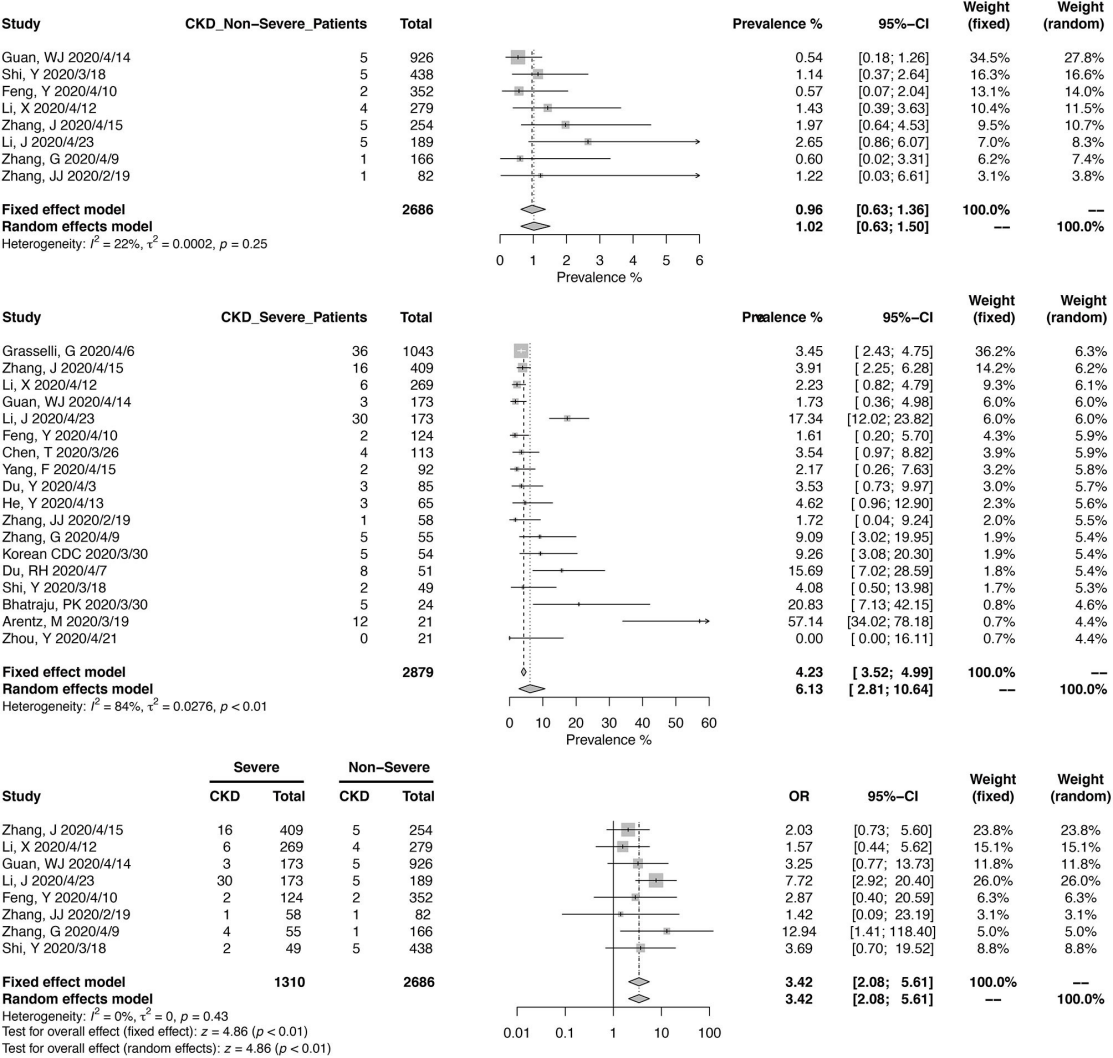
Correlation between chronic kidney disease and disease severity in COVID-19 patients.

Eight studies, including 2,686 Chinese patients, were included in the meta-analysis of CKD prevalence in non-severe COVID-19 (Figure 3). The pooled prevalence was 1.02% (95% CI, 0.63–1.50%), with mild heterogeneity noted (*I*^2^ = 22%, *p* = 0.25). For severe COVID-19 cases, pooled CKD prevalence from 2,879 patients (18 studies) was 6.13% (95% CI, 2.81–10.64%). A meta-analysis from eight studies, reporting 1,310 severe and 2,686 non-severe cases, found that COVID-19 patients with CKD could have a higher risk of developing into severe cases (OR, 3.42; 95% CI, 2.05–5.61; heterogeneity *I*^2^ = 0%, *p* = 0.43).

**Figure 4:**
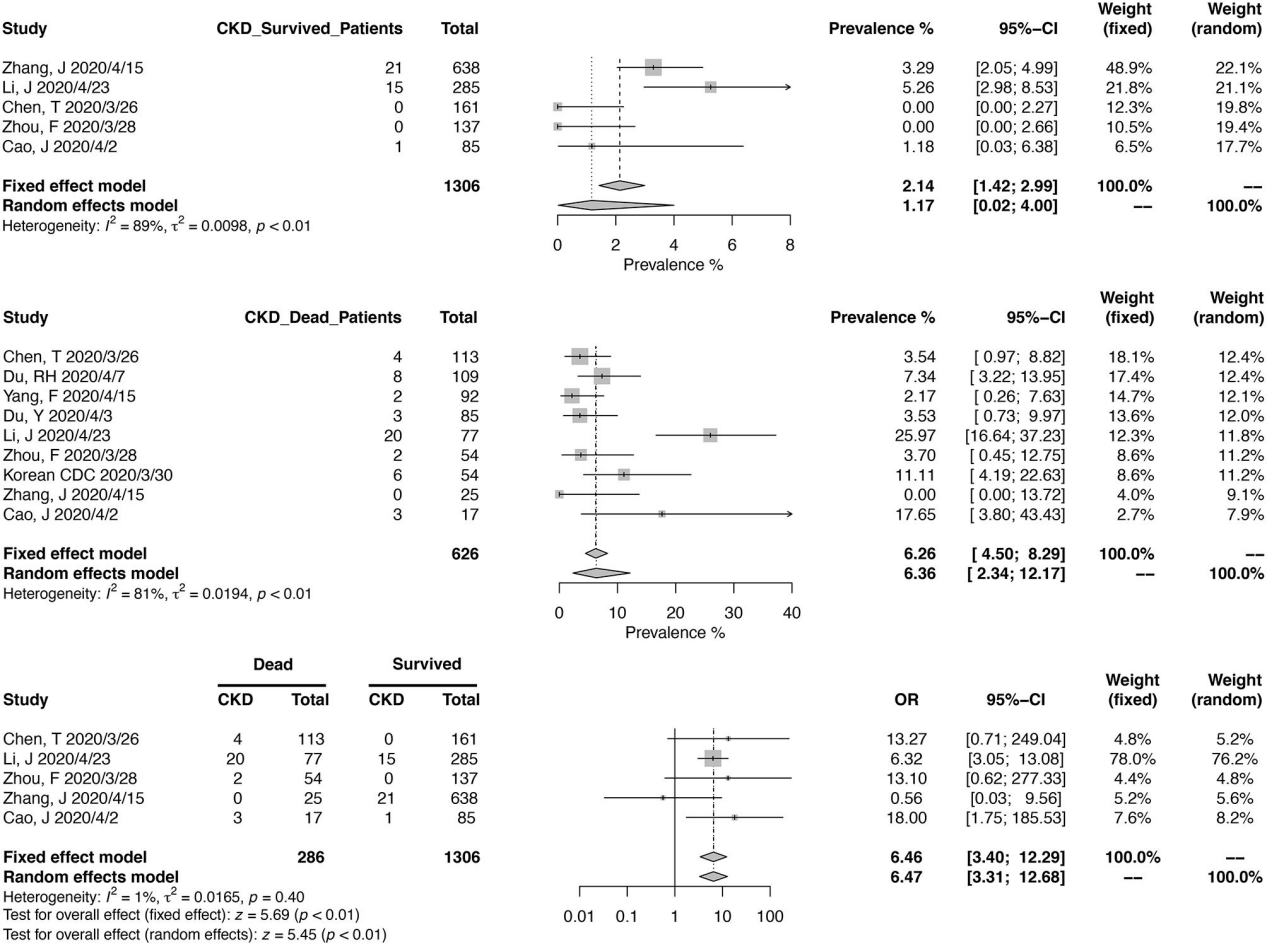
Correlation between chronic kidney disease and mortality in COVID-19 patients.

Similarly, we calculated the pooled CKD prevalence in survived (1.17%; 95% CI, 0.02–4.00%) and deceased patients (6.36%; 95% CI, 2.34–12.17%) (Figure 4). The heterogeneities were both significant in these two analyses (*I*^2^ > 75%, *p* < 0.01). The mortality risk was increased in CKD patients (OR, 6.46; 95% CI, 3.40–12.29; heterogeneity *I*^2^ = 1%, *p* = 0.40), based on the analysis from 1,306 survived and 286 deceased patients (Figure 4).

### Prevalence of Acute Kidney Injury and Associations With Prognosis

**Figure 5:**
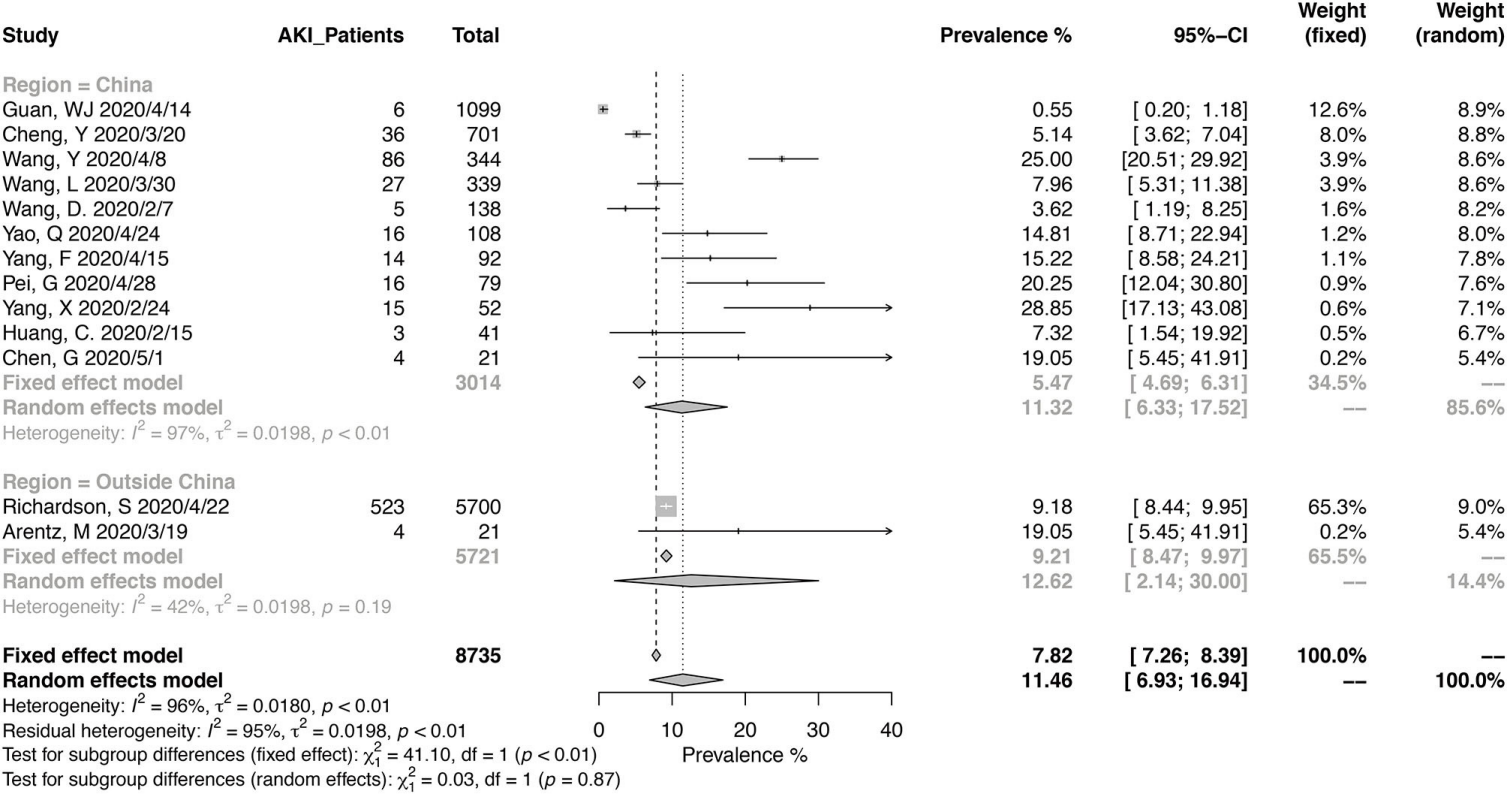
Pooled prevalence of acute kidney injury in COVID-19 patients.

**Figure 6:**
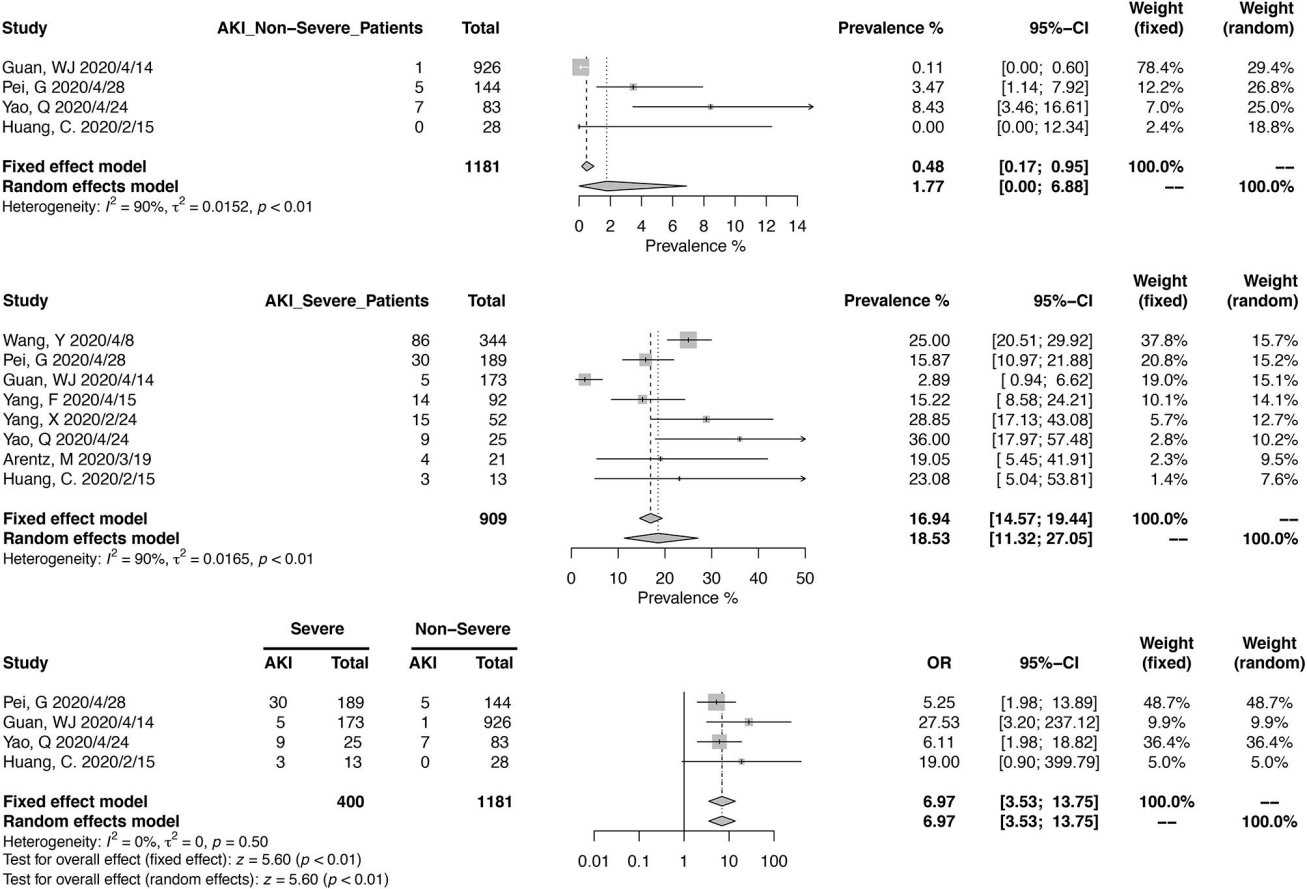
Correlation between acute kidney injury and disease severity in COVID-19 patients.

**Figure 7:**
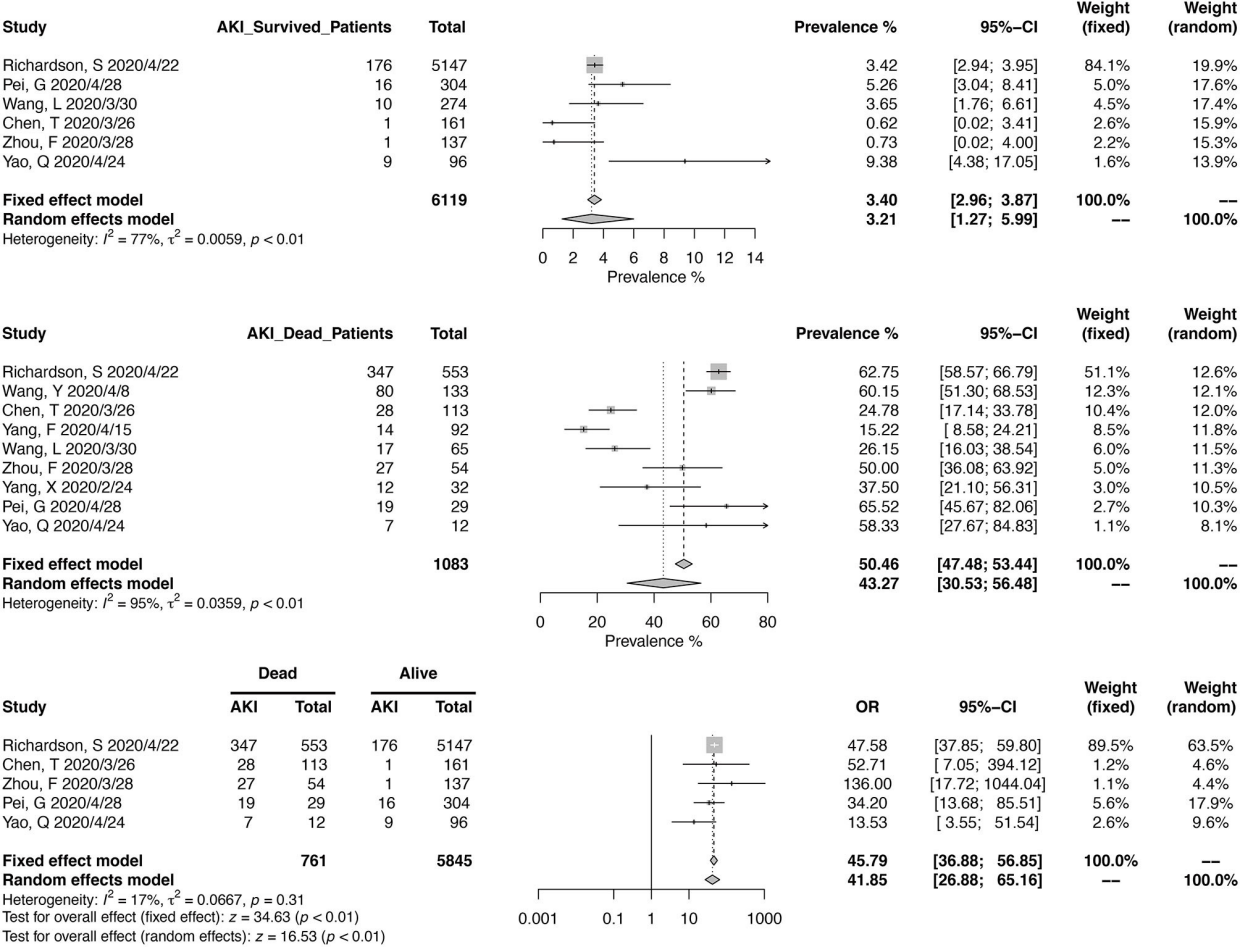
Correlation between acute kidney injury and mortality in COVID-19 patients.

Thirteen studies have reported the prevalence in AKI in a total of 8,735 patients, with diagnostic criteria stated in the manuscripts (Figure 5). The summary estimates for AKI prevalence was 11.46% (95% CI, 6.93–16.94%), which was similar between Chinses studies and data from other countries. The pooled prevalences of AKI were 1.77% (95% CI, 0.00–6.88%) in non-severe patients, 18.53% (95% CI, 11.32–27.05%) in severe patients, 3.21 (95% CI, 1.27–5.99%) in survived patients, and 43.27% (95% CI, 30.53–56.48%) in deceased ones (Figures 6, 7). Significant heterogeneity for AKI prevalence was seen across studies, including those in the subgroup analyses (*I*^2^ > 75%, *p* < 0.01). The risk of deterioration was significantly higher in patients with AKI (OR, 6.97; 95% CI, 3.53–13.75; heterogeneity *I*^2^ = 0%, *p* = 0.50, Figure 6) and so was the mortality risk (OR, 45.79; 95% CI, 36.88–56.85; heterogeneity *I*^2^ = 17%, *p* = 0.31, Figure 7).

### Preprint Studies and Other Comorbidities

We performed the same estimation based on publications in the preprint platforms (Supplementary Figures 1, 2). The pooled prevalences were 3.17% (95% CI, 2.15–4.39%) for CKD and 7.22% (95% CI, 2.95–13.19%) for AKI, and neither was significantly different compared to those from the peer-reviewed data (*p* > 0.05). The heterogeneity was significant for both (*I*^2^ > 75%, *p* < 0.01).

We estimated the prevalence of other comorbidities in the peer-reviewed publications (Supplementary Figures 3–7). The summary estimates for the prevalence of hypertension was 25.96% (95% CI, 21.15–31.08%), diabetes mellitus was 13.98% (95% CI, 11.04–17.20%), cardiovascular disease was 9.85% (95% CI, 6.66–13.59%), the cerebrovascular disease was 3.74% (95% CI, 2.09–5.85), and malignancy was 2.99% (95% CI, 2.18–3.92%). Significant heterogeneity was noted among all these studies (*I*^2^ > 75%, *p* < 0.01). The prevalences of hypertension, diabetes mellitus, and cardiovascular disease were significantly higher than that of CKD (*p* < 0.05).

### Assessment for Publication Bias

The risk of publication bias was analyzed by the funnel plots and Eggers test, which suggested no significant publication bias (Supplementary Figure 8, all *p* > 0.05).

## Discussion

The global prevalence of CKD was estimated to be 9.1% recently (12), but a paucity of data was noted concerning the risk of these people in the COVID-19 pandemic. In this meta-analysis, we addressed the prevalence of kidney diseases in COVID-19 patients and their impacts on adverse disease courses based on the available reports. The presence of kidney diseases, in the forms of CKD and AKI, tended to develop into more severe cases and was associated with increased mortality. However, the presence of both CKD and AKI identified throughout the included cohorts seemed disproportionately scarce compared to data revealed in previous epidemiology studies (12).

Several reasons are urging us to emphasize the importance of CKD during the COVID-19 infection. First, CKD has not attracted enough awareness due to its inconspicuous course, especially in the early stage (13, 15, 64). Second, diabetes and hypertension are the leading causes of CKD in all developed countries and many developing countries, and the long-term or advanced CKD usually increases the risk of cardiovascular diseases (13, 65). To be noted, these conditions accompanying CKD are all risk factors that exacerbate the COVID-19 patients (10, 11). Third, glomerulonephritis is another relevant CKD entity, and patients falling into this category usually take immunosuppressive medicine (13). Some early reports suggested that patients with a compromised immune system, such as patients with advanced CKD or those undergoing immunosuppressives, hypothetically limited the cytokine strome in the COVID-19 infection and led to mild disease courses (66–70). This was not the case in our investigation. Either in terms of disease severity or mortality, we found that CKD unfavorably impacted the COVID-19 infection. Fourth, preexisting CKD is a significant risk factor for worsening of kidney function during a severe infection (71, 72). Once complicated by AKI, CKD itself was associated with a higher risk of mortality and less chance of kidney recovery (73).

Although far from detailed, the importance of AKI has been more profiled by several reports in COVID-19 patients (6, 7). More recently, several meta-analyses have proved the important prognostic values of AKI in severe COVID-19 patients (74–77). As a vital complication, the presence of AKI was associated with more adverse prognosis in the disease course. This was consistent with our findings in the current meta-analysis, which has signaled dissatisfied results associated with AKI. However, the prevalence of AKI ranged from 4 to 17% in these meta-analyses. The diagnosis criteria of AKI was not clarified in all meta-analysis, neither was the diagnosis of severe COVID-19 cases. We only included the studies referring to the Kidney Disease: Improving Global Outcomes (KDIGO) criteria of AKI diagnosis. The overlap of patients in the included studies was not addressed in these meta-analyses, which could promote bias. Moreover, the etiologies of AKI, another substantial issue (78), have not been thoroughly investigated in the current literature. Multiple factors may lead to AKI in the COVID-19 setting, such as the potential virus insulation in the kidney tissues (45), inflammatory factors (67), hemodynamic changes, volume depletion, and drug-induced damages. It is essential to respect the causes of different disease periods since they are the keys to improve kidney function and disease prognosis.

In reflection of our findings in this meta-analysis, we genuinely feel that our current prevention, control, and treatment measures for the COVID-19 have not yet been personalized. People with kidney disease have limited voice in this disease with lung-dominated involvement. As the pandemic persists, more precise prevention policies may be needed to guide patients with chronic kidney diseases, such as social distancing. Close monitoring of suspected symptoms for early recognition and interventions are important in the regular follow-ups of these patients. As in treating patients with severe COVID-19, the prevention and optimized management of AKI might help improve the prognosis, which is based on the notion that suspected intrinsic AKI accounted for the most frequent form of AKI (>80%) (6, 7). Risk factors and causes of AKI in COVID-19 are diverse and multifactorial, while severe hypoxia and hypercoagulability are the most important ones. It is necessary for intensive supporting and careful monitoring of patients with severe and critically ill pneumonia to ameliorate renal complications.

Therefore, there are some limitations to this study. First, this meta-analysis was mainly derived from retrospective cohort studies during a short period, which impaired the quality of data in several ways. A majority of studies were from China, and duplication reports of individual patients existed. We used NOS scores to evaluate the quality of included studies and matched the studies by the locations and recruitment time points to minimize the duplication in the pooled analysis. Second, the meta-analysis of prevalence was intrinsically heterogeneous, as shown in our results. Even though we performed the arcsine transformation, the heterogeneity was still significant for most of our prevalences. Third, the underreporting of CKD and AKI prevalence was a concern. Compared to hypertension and diabetes mellitus, the prevalence of CKD was disproportionally low. Meanwhile, the accurate diagnosis of AKI requires close monitoring of renal functions according to guidelines (78, 79), and defining the causes of AKI needs care differential diagnosis. Limited data were provided for further exploration. Fourth, it is difficult to profile specific confounding risk factors between renal insults and COVID-19 prognosis, based on the literature available until now. Although there is some inherited limitation in the meta-analysis, it signals some critical aspects of renal effects associated with the COVID-19 infection, providing clues for further well-designed researches.

With this meta-analysis of updated data, we found that CKD and AKI were all associated with worse prognosis of COVID-19. Patients with CKD should hence be advised to take extra precautions to minimize exposure to the virus. Physicians should also be engaged in close monitoring of COVID-19 patients with preexisting or new kidney involvements. Finally, the presence of CKD or AKI shall be regarded as essential factors in future risk stratification models for COVID-19.

## Methods

### Search Strategy

A systematic review of the literature was performed according to the Preferred Reporting Items for Systemic Reviews and Meta-Analyses (PRISMA) statement. Publicly available publication list of COVID-19 living systematic review was retrieved up to April 26, 2020, containing studies on COVID-19 published on PubMed, Embase (via Ovid), bioRxiv, and medRxiv in a daily updated manner (80). We validated the list by manually searching relevant studies in the databases above (details in Supplementary Table 1). Additionally, we included studies that were available publicly but not on the list at the time of the search (6).

### Study Selection

All studies were considered without limitations on language or publication status (peer-reviewed or preprint). Titles and abstracts were first reviewed by the investigators (YZ and QJ), and potential COVID-19-related papers on clinical characteristics were retrieved. Duplicate studies and those reporting one patient were removed based on titles. Two investigators (QR and GC) independently determined the eligibility of studies, and dissonance was resolved through discussion with the third investigator (YZ). The inclusion criteria included the following: (1) study populations were adult COVID-19 patients with virologic proof and (2) study designs included case series, cohort studies, case–control studies, and randomized controlled trials. The exclusion criteria were the following: (1) review articles, meta-analyses, editorials or comments, and summaries; (2) studies that only report pediatric patients or one patient; (3) studies that did not report CKD and/or AKI data; and (4) studies that the diagnostic criteria of AKI were not defined.

### Data Extraction and Quality Assessment

At least two independent reviewers (from YZ, QR, GC, HL, and QC) extracted the following information, including first authors, inclusion/exclusion criteria, patient's location and recruitment date, sample size, age, sex, disease severity, any morbidities (CKD, hypertension, diabetes mellitus, cardiovascular diseases, cerebrovascular diseases, or malignancies), presence of AKI, and death. Disease severity was defined according to the guideline from the American Thoracic Society and Infectious Disease Society (81), Chinese COVID-19 management guideline (82), need of ICU admission, or death. The diagnosis references of AKI were collected if available, including the 2012 KDIGO definition (78, 79) or others.

The NOS was used to evaluate the quality of enrolled studies in terms of patient selection, comparability, and results (83). Each study was scored according to the exposure (CKD or AKI) and endpoint (disease severity or prognosis) individually. The quality of the included studies was assessed independently by two independent reviewers (from YZ, QR, and GC). NOS scores of at least six were considered high-quality literature.

### Data Synthesis and Statistical Analyses

To assess the prevalence of CKD or AKI and their relationship with COVID-19 disease courses, the meta-analysis was prespecified to be conducted for all the infected individuals and based on patient stratifications: (1) disease severities (mild or severe) and (2) clinical outcomes of COVID-19 (dead or survived). Studies from the same healthcare facilities were reviewed by three investigators (YZ, QR, and GC), and duplicate reports were carefully excluded.

Continuous variables were expressed as median [interquartile range (IQR)] or mean [±standard deviation (SD)]. The prevalence of CKD or AKI was expressed as proportion and 95% confidence interval (95% CI) using the random-effects model. Odds ratios (ORs) with 95% CI were calculated in evaluating the risk of severe diseases or death in patients with CKD or AKI separately. Heterogeneity among studies was detected with the Cochrane's Q-test, and a *p* < 0.05 was considered as significant heterogeneity. The *I*^2^ statistic was performed to evaluate the contribution of heterogeneity in the overall study variation. τ^2^ was calculated to estimate the between-study variance using the Paule–Mandel method, and the 95% CI was calculated using the Q-Profile method. Subgroup analysis was performed on the patients' locations (China or outside of China), disease severities (mild or severe), and prognosis (dead or survived). The Q test for heterogeneity was used to test the significance of the overall between-groups variance with the assumption of the shared common τ^2^ across subgroups. All statistics were performed using the meta package (Version 4.11-0) in the R program (Version 3.6.3. R core team). We used arcsine transformation and inverse variance method to implement the calculation of the overall prevalence of CKD or AKI, and the confidence interval was calculated with the Clopper–Pearson method. All data shown in Forrest plots and results section was back transformed to the raw prevalences.

## Data Availability Statement

The raw data supporting the conclusions of this article will be made available by the authors, without undue reservation.

## Author Contributions

YZ, QR, and GC were responsible for study design, literature research, data extraction, data synthesis, and manuscript drafting. QJ, QC, and HL helped in data extraction. KZ and YQ helped in study design and manuscript revision. XL were responsible for study design, organizing, and manuscript revision. All authors contributed to the article and approved the submitted version.

## Conflict of Interest

The authors declare that the research was conducted in the absence of any commercial or financial relationships that could be construed as a potential conflict of interest.
