# Supplementary material for: Chronic Kidney Diseases and Acute Kidney Injury in Patients With COVID-19: Evidence From a Meta-Analysis
Source: Front Med (Lausanne). 2020 Nov 3;7:588301. doi: 10.3389/fmed.2020.588301 (PMC7670057; doi:10.3389/fmed.2020.588301)
Supplement: Supplementary file 1 [file Table_1.docx]

Supplementary Table.1 Search terms

| PubMed | ("COVID-19" OR “SARS-CoV-2” OR "2019 ncov"[tiab] OR (("novel coronavirus"[tiab] OR "new coronavirus"[tiab]) AND (wuhan[tiab] OR 2019[tiab])) OR 2019-nCoV[All Fields] OR (wuhan[tiab] AND coronavirus[tiab])) |
| --- | --- |
| EMBASE | (nCoV or 2019-nCoV or ((new or novel or wuhan) and coronavirus) or covid19 or covid-19 or SARS-CoV-2) |
| BioRxiv/MedRxiv | (ncov or corona or wuhan or COVID or SARS-CoV-2) |
