# Supplementary material for: Chronic Kidney Diseases and Acute Kidney Injury in Patients With COVID-19: Evidence From a Meta-Analysis
Source: Front Med (Lausanne). 2020 Nov 3;7:588301. doi: 10.3389/fmed.2020.588301 (PMC7670057; doi:10.3389/fmed.2020.588301)
Supplement: Supplementary file 2 [file Table_2.docx]

Supplementary Table.2 Characteristics of the studies included for meta-analysis

|  |  |  |  |  |  |  |  |  |  |  | **Severity** | | | | **CKD (n, %)** | | | | **AKI (n, %)** | | | | **AKI definition** |
| --- | --- | --- | --- | --- | --- | --- | --- | --- | --- | --- | --- | --- | --- | --- | --- | --- | --- | --- | --- | --- | --- | --- | --- |
| **Study** | **PMID** | **Publish Date** | **Journal** | **Country** | **City/Province** | **Sample Size** | **Sample Source** | **Recruitment period** | **Recruitment method** | **NOS scores** | **Severe definition** | **Severe** | **Non-Severe** | **Death** | **All** | **Severe** | **Non-Severe** | **Death** | **All** | **Severe** | **Non-Severe** | **Death** |  |
| Wang, D. | 32031570 | 2020/2/7 | JAMA | China | Wuhan/Hubei | 138 | Zhongnan Hospital of Wuhan University | 1 Jan-28 Jan | consecutive | 6 | ICU admission | 36 | 102 | 6 | 4 | 2 | 2 |  | 5 | 3 | 2 |  | 2012-KDIGO |
| Liu, Y. | 32048163 | 2020/2/9 | Sci China Life Sci | China | Shenzhen/Guangdong | 12 | Shenzhen Third People’s Hospital | 11 Jan-20 Feb | consecutive | ≥6 |  |  |  |  | 4 |  |  |  |  |  |  |  |  |
| Huang, C. | 31986264 | 2020/2/15 | Lancet | China | Wuhan/Hubei | 41 | Jinyintan Hospital | To 2 Jan | consecutive | 6 | ICU admission | 13 | 28 | 6 |  |  |  |  | 3 | 3 | 0 |  | 2012-KDIGO |
| Zhang, JJ | 32077115 | 2020/2/19 | Allergy | China | Wuhan/Hubei | 140 | No.7 hospital of Wuhan | 16 Jan-3 Feb | consecutive | 6 | Chinese guideline | 58 | 82 |  | 2 | 1 | 1 |  |  |  |  |  |  |
| Xu, XW | 32075786 | 2020/2/19 | BMJ | China | Zhejiang | 62 | 7 local designated hospitals | 10 Jan-26 Feb | consecutive | ≥6 | ICU admission | 1 | 61 | 0 | 1 |  |  |  |  |  |  |  |  |
| Yang, X | 32105632 | 2020/2/24 | Lancet Respir Med | China | Wuhan/Hubei | 52 | Wuhan Jin Yin-tan hospital | To 26 Jan | ICU patients | ≥6 | ICU admission | 52 |  | 32 |  |  |  |  | 15 | 15 |  | 12 | 2012-KDIGO |
| Guan, WJ | 32109013 | 2020/4/14 | N Engl J Med | China | National | 1099 | 552 hospitals in 30 provinces | To 29 Jan | consecutive | ≥6 | American Thoracic Society guidelines | 173 | 926 | 15 | 8 | 3 | 5 |  | 6 | 5 | 1 |  | 2012-KDIGO |
| Wu, C | 32167524 | 2020/3/13 | JAMA Intern Med | China | Wuhan/Hubei | 201 | Jinyintan Hospital | 25 Dec-26 Jan | consecutive | ≥6 | ARDS | 84 | 117 | 44 | 2 |  |  |  |  |  |  |  |  |
| Zhou, F | 32171076 | 2020/3/28 | Lancet | China | Wuhan/Hubei | 191 | Jinyintan Hospital, Wuhan Pulmonary Hospital | To 31 Jan | consecutive | ≥6 | Chinese guideline | 119 | 72 | 54 | 2 |  |  | 2 | 28 |  |  | 27 | 2012-KDIGO |
| Liu, K | 32171866 | 2020/3/27 | J Infect | China | Hainan | 56 | Hainan General Hospital | 1 Jan-15 Feb | consecutive | ≥6 |  |  |  | 3 | 1 |  |  |  | 10 |  |  |  | NA |
| Arentz, M | 32191259 | 2020/3/19 | JAMA | US | Washington | 21 | Evergreen Hospital | 20 Feb-5 Mar | consecutive | ≥6 | ICU admission | 21 |  | 11 | 12 | 12 |  |  | 4 | 4 |  |  | 2012-KDIGO |
| Shi, Y | 32188484 | 2020/3/18 | Crit Care | China | Zhejiang | 487 | The First Affiliated Hospital of Zhejiang University | To 17 Feb | consecutive | 6 | NA | 49 | 438 | 0 | 7 | 2 | 5 |  |  |  |  |  |  |
| Sun, Y | 32211755 | 2020/3/25 | Clin Infect Dis | Singapore | Singapore | 54 | National Centre for Infectious Diseases | 26 Jan-16 Feb | consecutive | ≥6 |  |  |  |  | 0 |  |  |  |  |  |  |  |  |
| Jin, X | 32213556 | 2020/3/24 | Gut | China | Zhejiang | 651 | Local designated hospitals | 17 Jan-8 Feb | consecutive | 6 | Chinese guideline | 64 | 587 | 1 | 6 |  |  |  |  |  |  |  |  |
| McMichael, TM | 32220208 | 2020/3/27 | N Engl J Med | US | Washington | 167 | A nursing facility | 28 Feb-18 Mar | consecutive | ≥6 |  |  |  | 35 | 43 |  |  |  |  |  |  |  |  |
| Guo, T | 32219356 | 2020/3/27 | JAMA Cardiol | China | Wuhan/Hubei | 187 | Seventh Hospital of Wuhan City | 23 Jan-23 Feb | consecutive | ≥6 |  |  |  | 43 | 6 |  |  |  | 18 |  |  |  | NA |
| Chen, G | 32217835 | 2020/5/1 | J Clin Invest | China | Wuhan/Hubei | 21 | Tongji Hospital | To 27 Jan | consecutive | 6 | Chinese guideline | 11 | 10 | 4 |  |  |  |  | 4 |  |  |  | 2012-KDIGO |
| Chen, T | 32217556 | 2020/3/26 | BMJ | China | Wuhan/Hubei | 274 | Tongji Hospital | 13 Jan-12 Feb | Deceased& Recovered | ≥6 |  |  |  | 113 | 4 |  |  | 4 | 29 |  |  | 28 | 2012-KDIGO |
| Bhatraju, PK | 32227758 | 2020/3/30 | N Engl J Med | US | Seattle | 24 | Local designated hospitals | 24 Feb-9 Mar | consecutive | ≥6 | ICU admission | 24 |  | 12 | 5 | 5 |  |  |  |  |  |  |  |
| Lescure, FX | 32224310 | 2020/3/27 | Lancet Infect Dis | France | Paris, Bordeaux | 5 | Local designated hospitals | To 24 Jan | consecutive | ≥6 | ICU admission | 3 | 2 | 1 | 0 |  |  |  | 1 |  |  |  | NA |
| Tang, X | 32224074 | 2020/3/26 | Chest | China | Wuhan/Hubei | 73 | Wuhan Pulmonary Hospital | 24 Dec-7 Feb | consecutive | ≥6 |  |  |  | 21 | 3 |  |  |  | 13 |  |  |  | NA |
| Korean CDC | 32233161 | 2020/3/30 | J Korean Med Sci | Korean | NA | 54 | National data | 19 Jan-10 Mar | Deceased | 6 | Death |  |  | 54 |  |  |  | 6 |  |  |  |  |  |
| Guo, W | 32233013 | 2020/3/31 | Diabetes Metab Res Rev | China | Wuhan/Hubei | 174 | Wuhan Union Hospital | 10 Feb-29 Feb | consecutive | ≥6 |  |  |  | 9 | 13 |  |  |  |  |  |  |  |  |
| Wang, L | 32240670 | 2020/3/30 | J Infect | China | Wuhan/Hubei | 339 | Renmin Hospital of Wuhan University | 1 Jan-6 Feb | Elderly | ≥6 |  |  |  | 65 | 13 |  |  | 4 | 27 |  |  | 17 | 2012-KDIGO |
| Cao, J | 32239127 | 2020/4/2 | Clin Infect Dis | China | Wuhan/Hubei | 102 | Zhongnan Hospital of Wuhan University | 3 Jan-1 Feb | consecutive | ≥6 |  |  |  | 17 | 4 |  |  | 3 | 20 |  |  | 15 | NA |
| Du, Y | 32242738 | 2020/4/3 | Am J Respir Crit Care Med | China | Wuhan/Hubei | 85 | Hannan Hospital, Wuhan Union Hospital | 9 Jan-15 Feb | Deceased | 6 |  |  |  | 85 |  |  |  | 3 |  |  |  |  |  |
| Kim, ES | 32242348 | 2020/4/6 | J Korean Med Sci | Korean | National | 28 | KNCCMC | Early Feb | consecutive | ≥6 |  |  |  | 0 | 0 |  |  |  |  |  |  |  |  |
| Lin, L | 32241899 | 2020/4/2 | Gut | China | Zhuhai/Guangdong | 95 | Fifth Affiliated Hospital of Sun Yat-sen University | 17 Jan-15 Feb | consecutive | ≥6 | WHO’s interim guidelines | 20 | 75 | 0 | 1 |  |  |  |  |  |  |  |  |
| Cheng, Y | 32247631 | 2020/3/20 | Kidney Int | China | Wuhan/Hubei | 701 | Tongji Hospital | 28 Jan-11 Feb | consecutive | ≥6 | Chinese guideline | 297 | 404 | 113 | 14 |  |  |  | 36 |  |  |  | 2012-KDIGO |
| Grasselli, G | 32250385 | 2020/4/6 | JAMA | Italy | Lombardy/Milan | 1591 | Fondazione IRCCS Ca’ Granda Ospedale Maggiore Policlinico | 20 Feb-18 Mar | ICU patients | ≥6 | ICU admission | 1591 |  | 405 | 36/1043 | 36/1043 |  |  |  |  |  |  |  |
| Du, RH | 32255382 | 2020/4/7 | Ann Am Thorac Soc | China | Wuhan/Hubei | 109 | Wuhan Pulmonary Hospital & Tianyou Hospital Affiliated to Wuhan Science and Technologgy University & Central Hospital of Wuhan | 25 Dec-15 Feb | Deceased | 6 | Death | 109 |  | 109 | 8 | 8 |  | 8 |  |  |  |  |  |
| Wang, Y | 32267160 | 2020/4/8 | Am J Respir Crit Care Med | China | Wuhan/Hubei | 344 | Tongji Hospital | 25 Jan-25 Feb | ICU patients | 6 | ICU admission | 344 |  | 133 |  |  |  |  | 86 | 86 |  | 80 | 2012-KDIGO |
| Liang, WH | 32269086 | 2020/4/8 | Eur Respir J | China | National | 1590 | 575 Hospitals in 31 Provinces | To 31 Jan | consecutive | ≥6 | American Thoracic Society guidelines | 131 | 1459 | 50 | 21 |  |  |  |  |  |  |  |  |
| Feng, Y | 32275452 | 2020/4/10 | Am J Respir Crit Care Med | China | Wuhan/Hubei,Shanghai,Anhui | 476 | Jinyintan Hospital in Wuhan, Shanghai Public Health Clinical Center in Shanghai, Tongling People's Hospital in Anhui Province | 1 Jan-15 Feb | consecutive | ≥6 | Chinese guideline | 124 | 352 | 38 | 4 | 2 | 2 |  |  |  |  |  |  |
| Chen, T | 32279081 | 2020/4/11 | J Gerontol A Biol Sci Med Sci | China | Wuhan/Hubei | 203 | Zhongnan Hospital of Wuhan University | 1 Jan-10 Feb | consecutive | ≥6 | Chinese guideline | 107 | 96 | 26 | 8 |  |  |  |  |  |  |  |  |
| He, Y | 32279676 | 2020/4/13 | Infect Control Hosp Epidemiol | China | Wuhan/Hubei | 65 | Tongji Hospital | 30 Dec-29 Feb | nosocomial infection patients | 6 | Chinese guideline | 65 |  | 10 | 3 | 3 |  |  |  |  |  |  |  |
| Li, X | 32294485 | 2020/4/12 | J Allergy Clin Immunol | China | Wuhan/Hubei | 548 | Sino-French New Town area Tongji Hospital | 26 Jan-5 Feb | consecutive | ≥6 | Chinese guideline | 269 | 279 | 90 | 10 | 6 | 4 |  | 95 | 62 | 33 |  | NA |
| Yang, F | 32293741 | 2020/4/15 | J Med Virol | China | Wuhan/Hubei | 92 | Renmin Hospital of Wuhan University | 6 Jan-25 Feb | Deceased | 6 | Death | 92 |  | 92 | 2 | 2 |  | 2 | 14 | 14 |  | 14 | 2012-KDIGO |
| Sun, C | 32295322 | 2020/4/15 | Zhonghua Jie He He Hu Xi Za Zhi | China | Nanyang/Henan | 150 | Local designated hospitals | 24 Jan-16 Feb | consecutive | ≥6 | Chinese guideline | 39 | 111 | 3 | 2 |  |  |  |  |  |  |  |  |
| Cholankeril, G | 32283101 | 2020/4/10 | Gastroenterology | USA | CA | 116 | Stanford University School of Medicine | 4 Mar-24 Mar 2020 | consecutive | ≥6 |  |  |  | 1 | 4 |  |  |  |  |  |  |  |  |
| Wang, X | 32302722 | 2020/4/14 | J Hosp Infect | China | Wuhan/Hubei | 80 | Tongji Hospital | 10 Jan-24 Feb | medical workers | 6 |  |  |  | 1 | 3 |  |  |  |  |  |  |  |  |
| Zhang, P | 32302265 | 2020/4/17 | Circ Res | China | Wuhan/Hubei | 1128 | Local designated hospitals | 31 Dec-20 Feb | HTN patients | 6 |  |  |  | 99 | 35 |  |  |  |  |  |  |  |  |
| Chen, R | 32304772 | 2020/4/15 | Chest | China | Nation Wide | 1590 | National Health Commission of the PRC (575 hospitals) | To 31 Jan | consecutive | ≥6 | ICU admission | 99 |  | 50 |  |  |  | 5 |  |  |  |  |  |
| Zhang, J | 32304745 | 2020/4/15 | Clin Microbiol Infect | China | Wuhan/Hubei | 663 | Renmin Hospital of Wuhan University | 11 Jan-5 Feb | consecutive | ≥6 | Chinese guideline | 409 | 254 | 25 | 21 | 16 | 5 | 0 | 68 | 56 | 12 | 5 | NA |
| Zhang, G | 32311650 | 2020/4/9 | J Clin Virol | China | Wuhan/Hubei | 221 | Zhongnan Hospital of Wuhan University | 2 Jan-10 Feb | consecutive | 6 | American Thoracic Society guidelines | 55 | 166 | 12 | 6 | 5 | 1 |  | 10 | 8 | 2 |  | NA |
| Zhou, Y | 32315487 | 2020/4/21 | Clin Transl Sci | China | Nanjing/Jiangsu | 21 | First Affiliated Hospital of Nanjing Medical University | 28 Jan-2 Mar | icu patients | 6 | Chinese guideline | 21 |  | 3 | 0 | 0 |  |  |  |  |  |  |  |
| Richardson, S | 32320003 | 2020/4/22 | JAMA | USA | New York City/NY | 5700 | 12 Northwell Health System Hospitals | 1 Mar-4 Apr | Consecutive | ≥6 |  |  |  | 553 | 454 |  |  |  | 523 |  |  | 347 | 2012-KDIGO |
| Li, J | 32324209 | 2020/4/23 | JAMA Cardiol | China | Wuhan/Hubei | 362 | Central Hospital of Wuhan | 15 Jan-15 Mar | Consecutive | 6 | Chinese guideline | 173 | 189 | 77 | 35 | 30 | 5 | 20 |  |  |  |  |  |
| Yao, Q | 32329978 | 2020/4/24 | Pol Arch Intern Med | China | Huanggang/Hubei | 108 | Dabieshan Medical Center | 30 Jan-11 Feb | Consecutive | ≥6 | American Thoracic Society guidelines | 25 | 83 | 12 |  |  |  |  | 16 | 9 | 7 | 7 | 2012-KDIGO |
| Yan, CH | 32329222 | 2020/4/24 | Int Forum Allergy Rhinol | USA | San Diego/CA | 128 | UC San Diego Health System (Jacobs and Hillcrest Medical Centers) | 3 Mar-8 Apr | Smell & Taste Patients | ≥6 |  |  |  | 1 | 4 |  |  |  |  |  |  |  |  |
| Kujawski, SA | 32327757 | 2020/4/23 | Nat Med | US | Six states | 12 | Local designated hospitals | 20 Jan-5 Feb | consecutive | 6 |  |  |  |  | 0 |  |  |  |  |  |  |  |  |
| Pei, G | 32345702 | 2020/4/28 | J Am Soc Nephrol | China | Wuhan/Hubei | 333 | Tongji Hospital | 28 Jan-9 Feb | Consecutive | ≥6 | Chinese guideline | 189 | 144 | 29 |  |  |  |  | 35 | 30 | 5 | 19 | 2012-KDIGO |

NOS, Newcastle-Ottawa Scale; CKD, chronic kidney disease; AKI, acute kidney injury.
