# Supplementary figures and images for: Chronic Kidney Diseases and Acute Kidney Injury in Patients With COVID-19: Evidence From a Meta-Analysis
Source: Front Med (Lausanne). 2020 Nov 3;7:588301. doi: 10.3389/fmed.2020.588301 (PMC7670057; doi:10.3389/fmed.2020.588301)

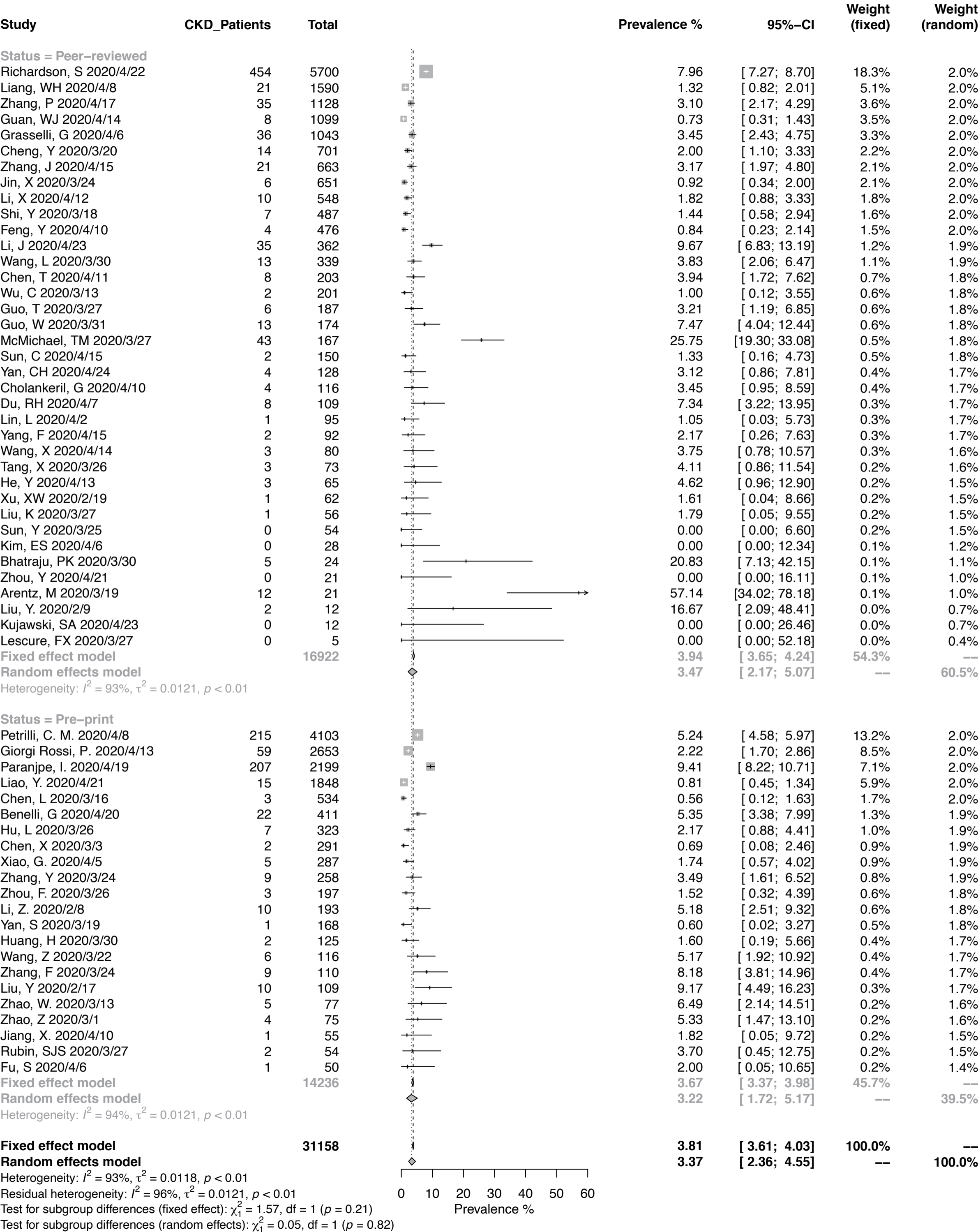

Supplement: Supplementary file 3 [file Data_Sheet_1.PDF]

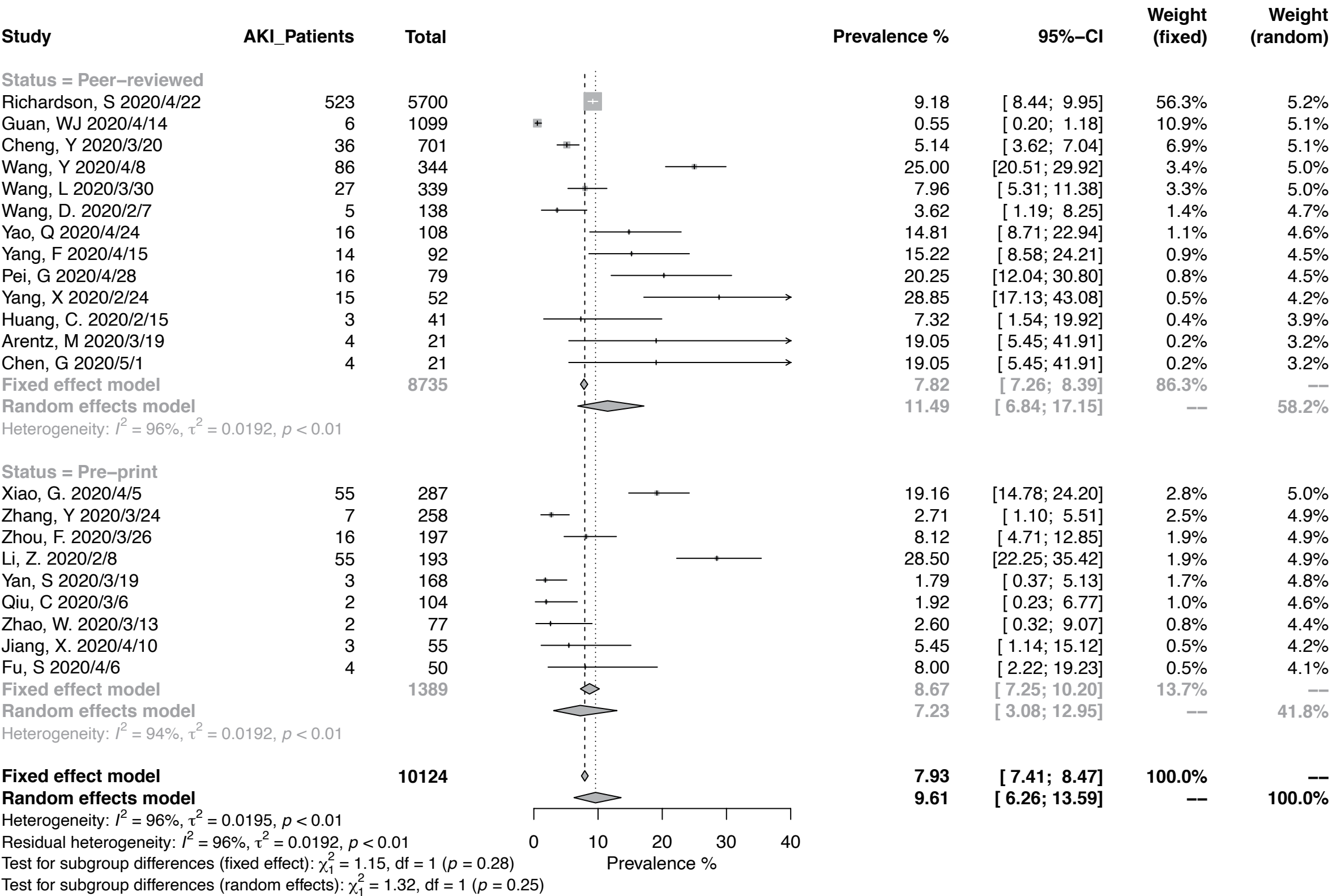

Supplement: Supplementary file 4 [file Data_Sheet_2.PDF]

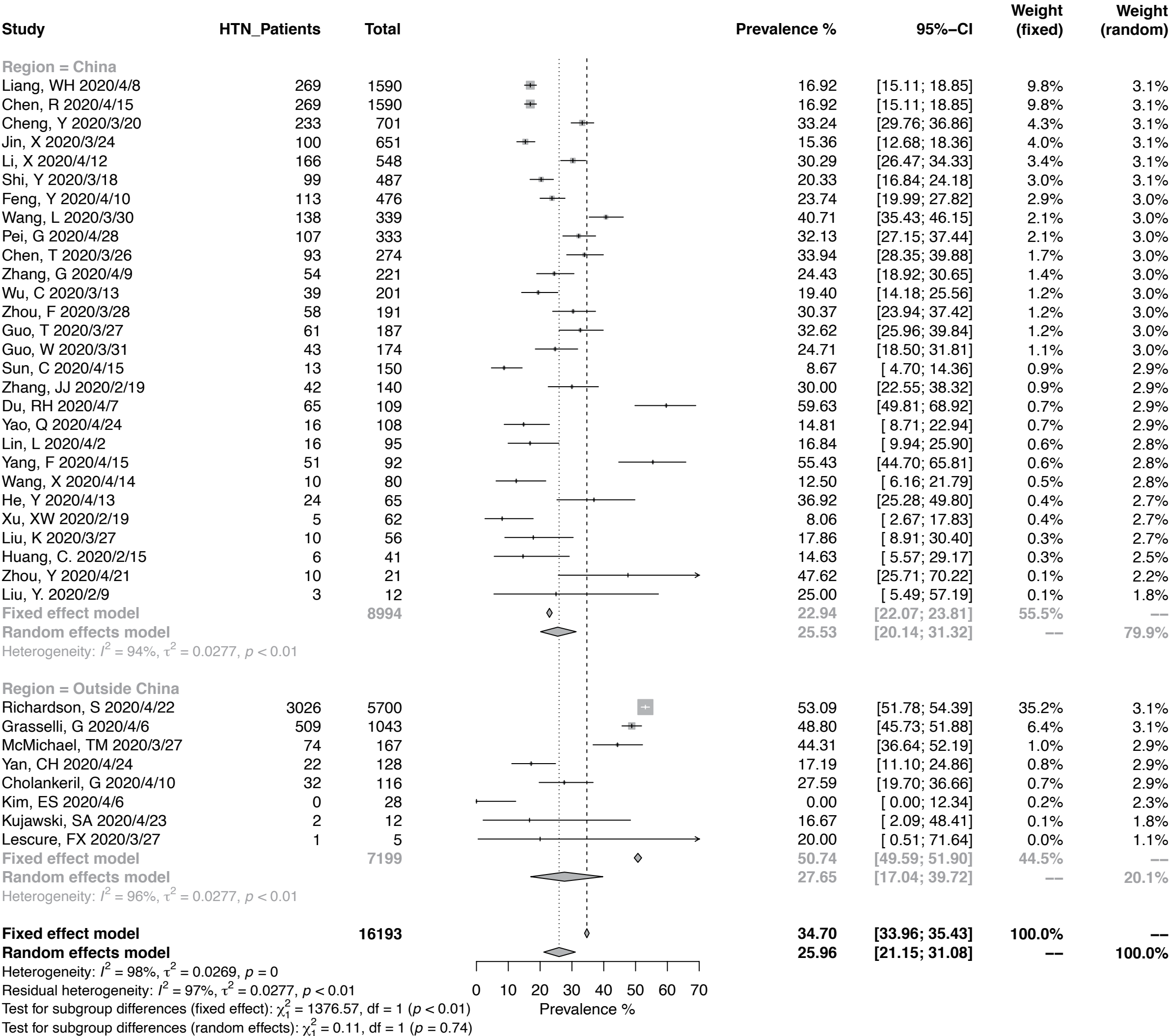

Supplement: Supplementary file 5 [file Data_Sheet_3.PDF]

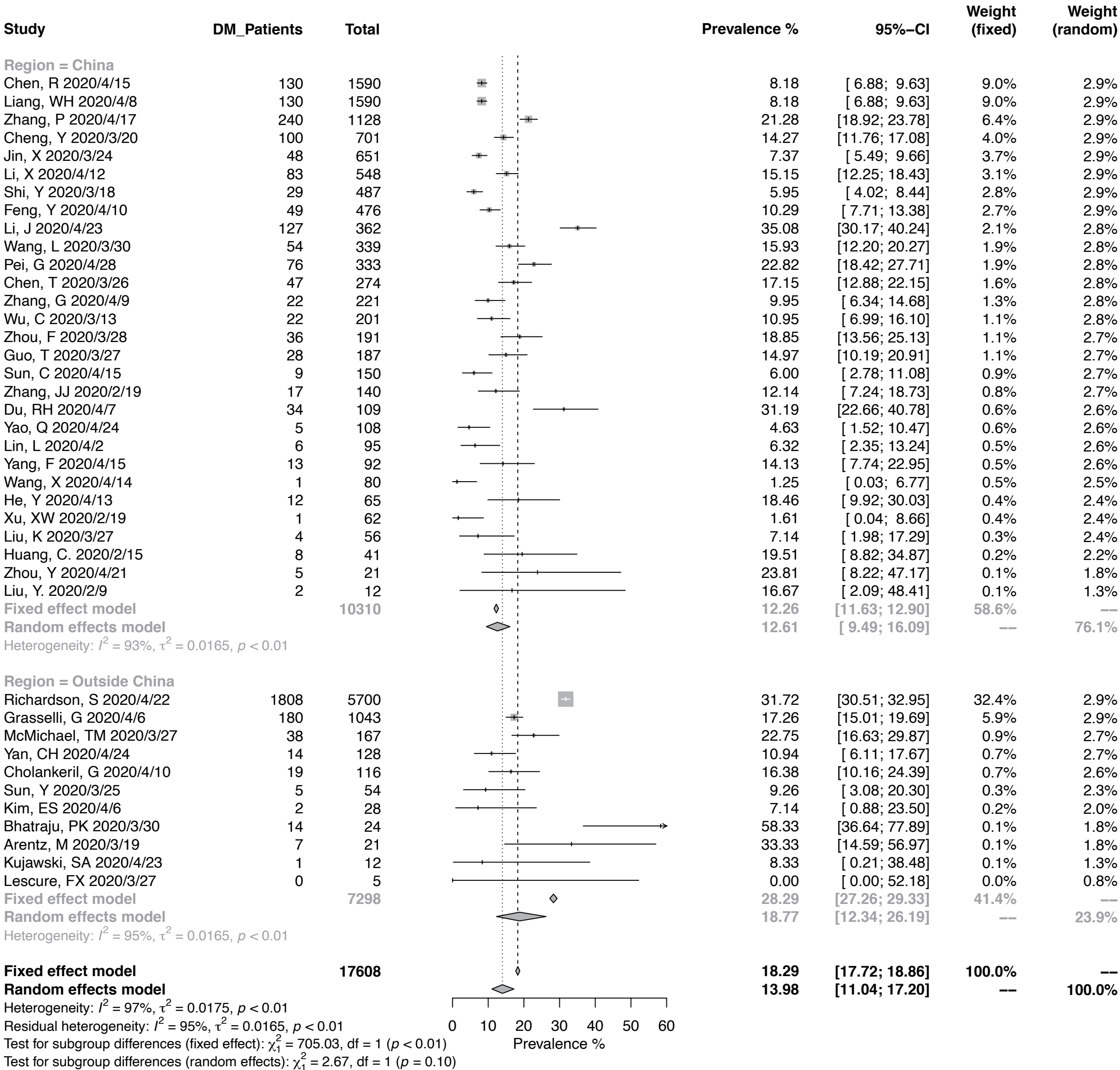

Supplement: Supplementary file 6 [file Data_Sheet_4.PDF]

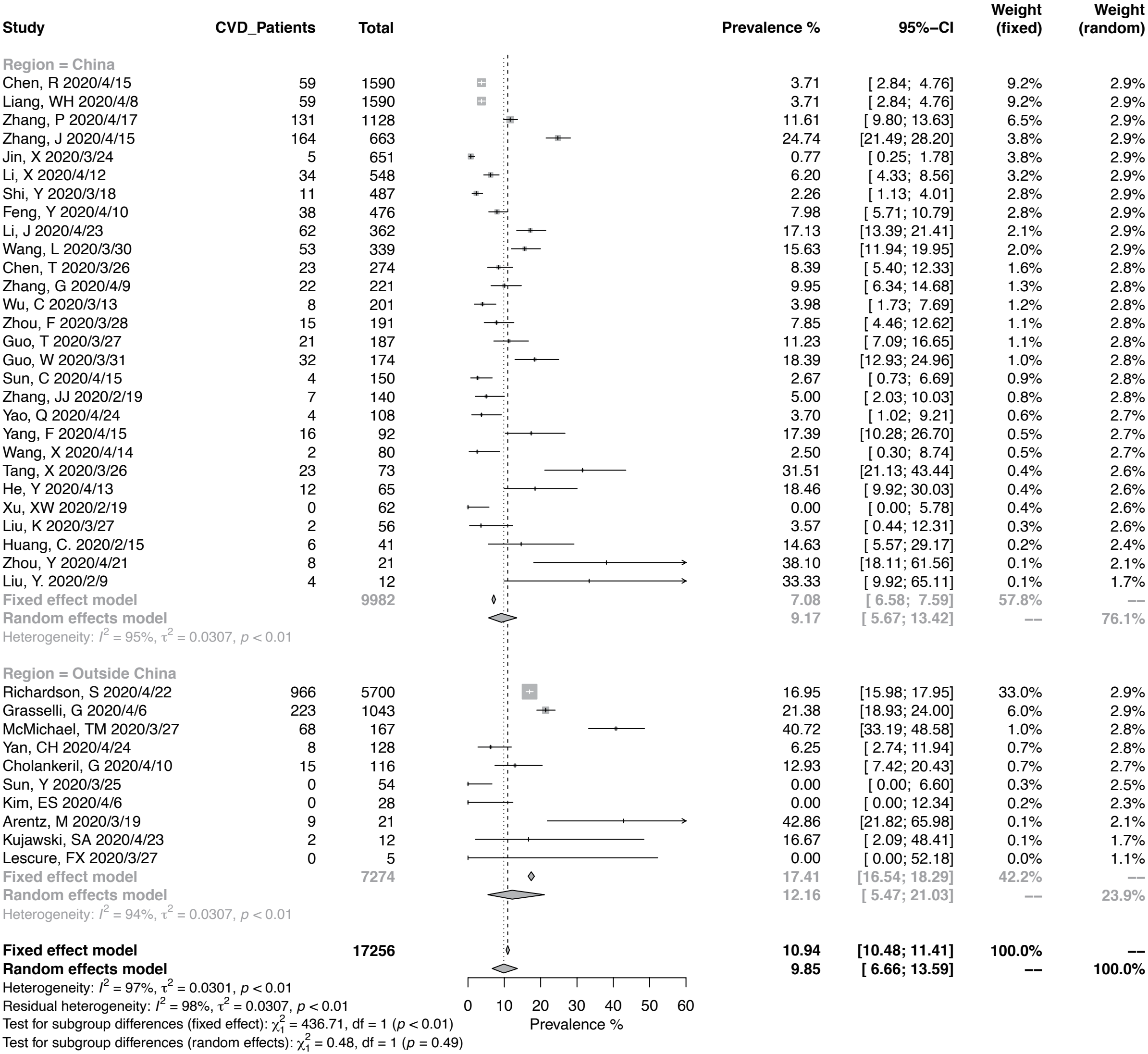

Supplement: Supplementary file 7 [file Data_Sheet_5.PDF]

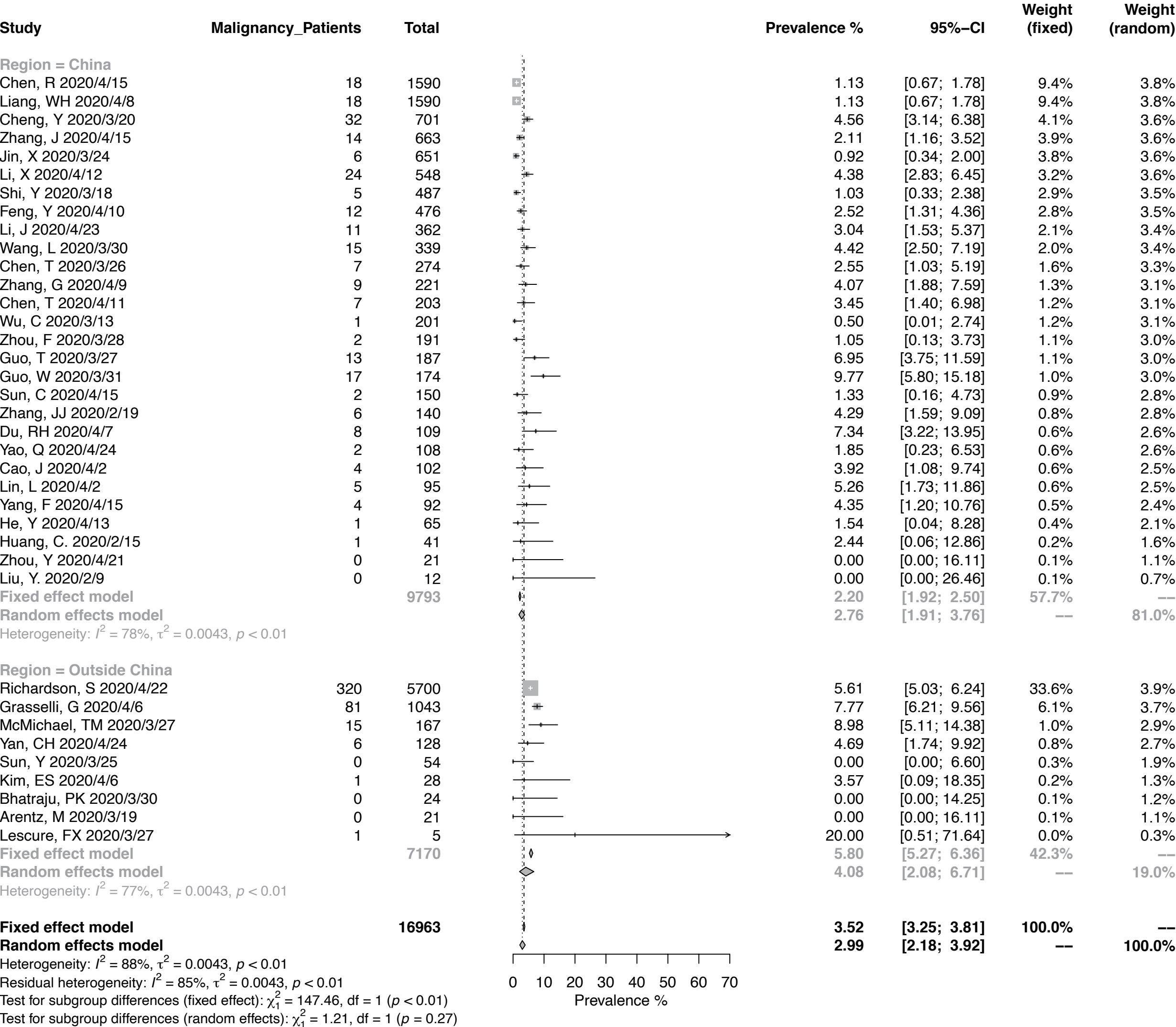

Supplement: Supplementary file 8 [file Data_Sheet_6.PDF]

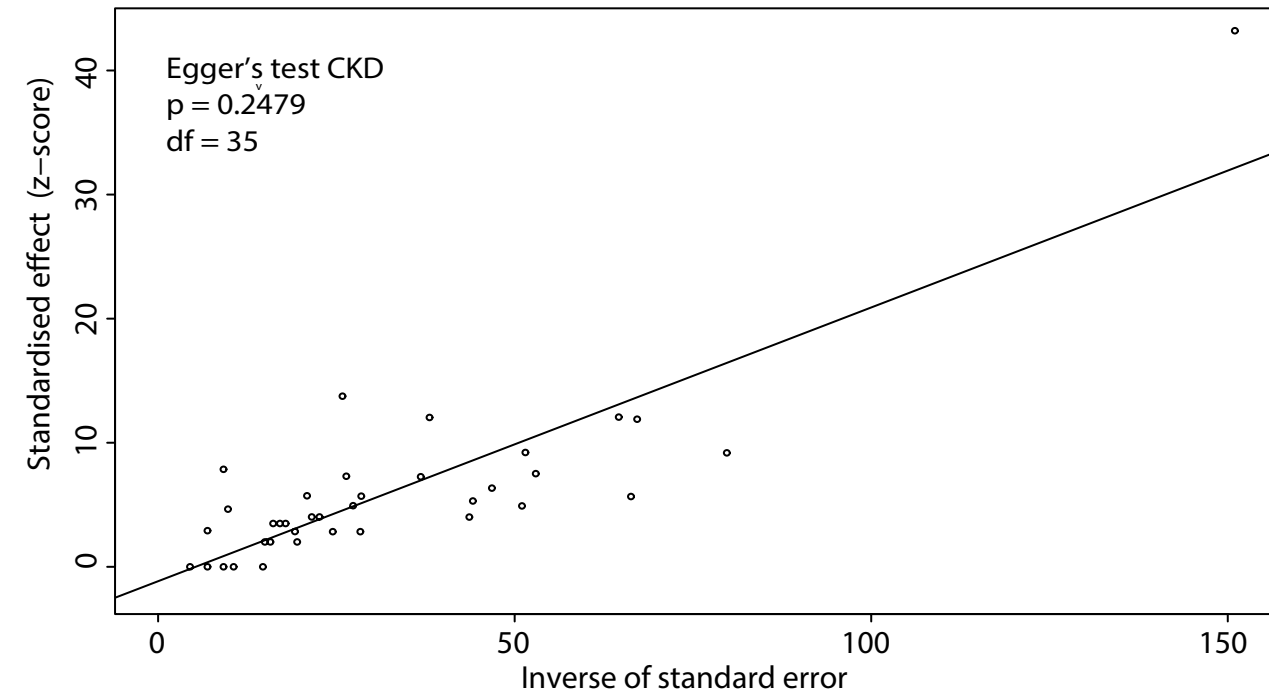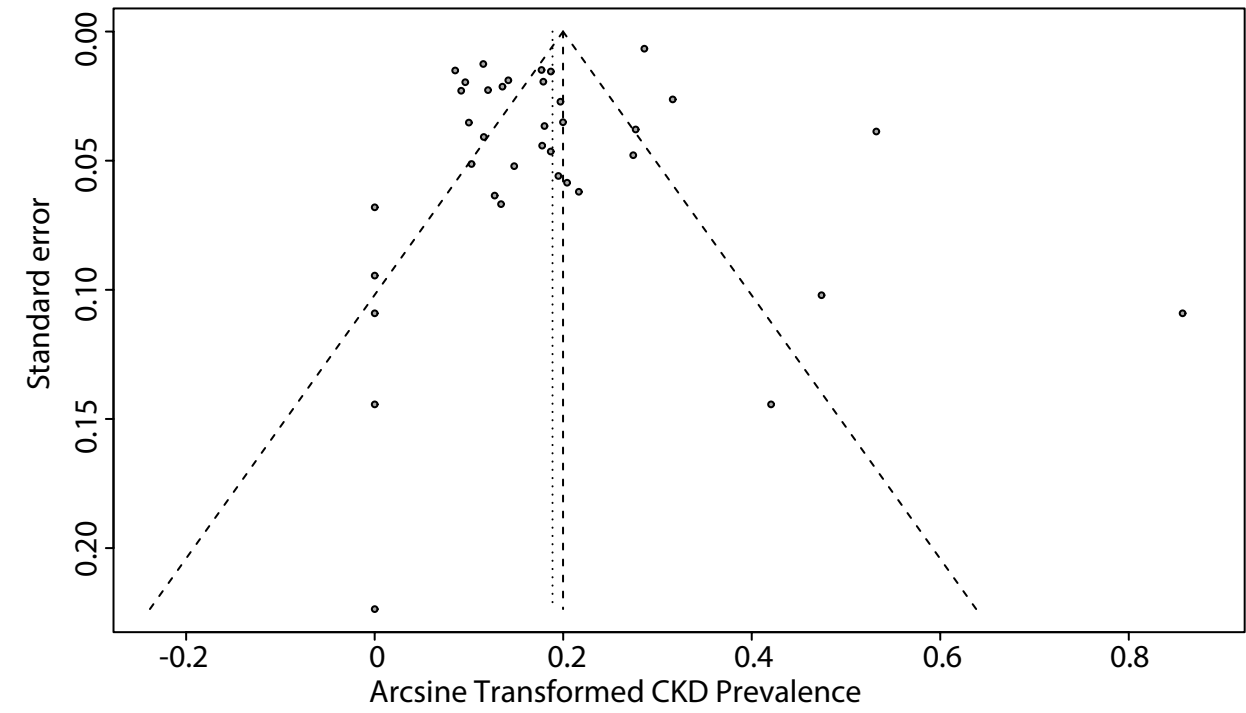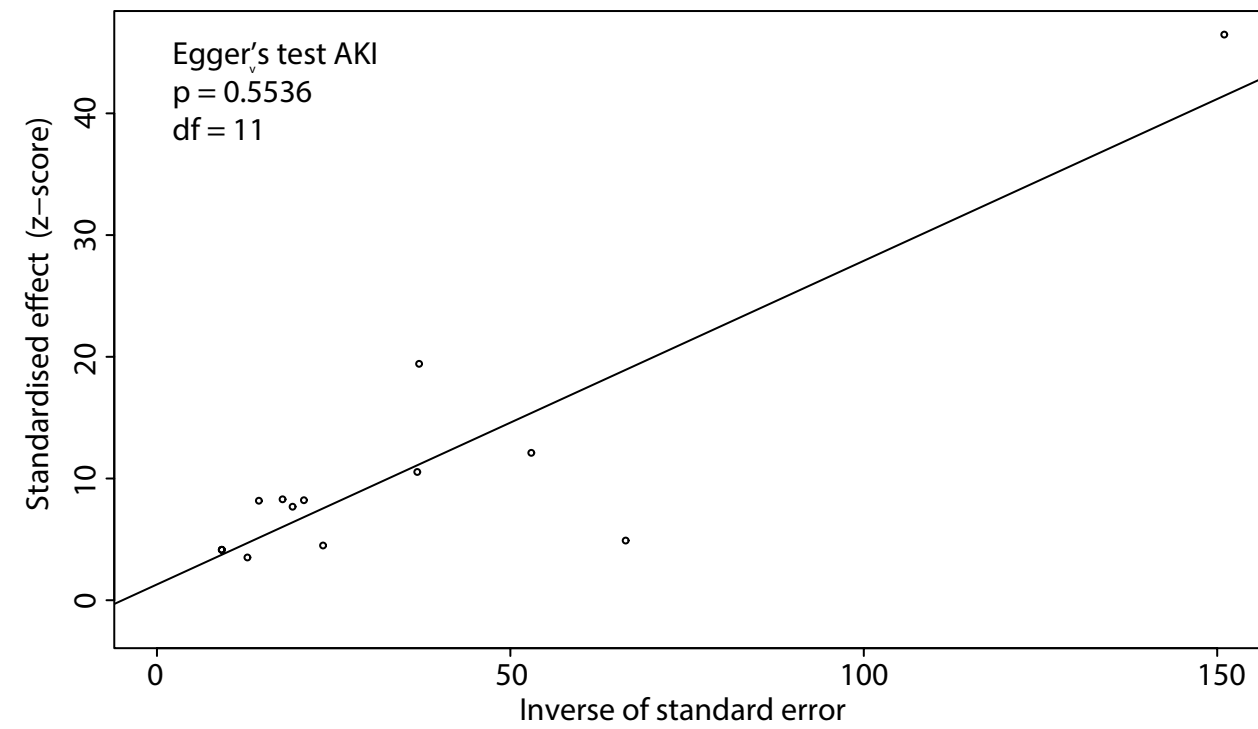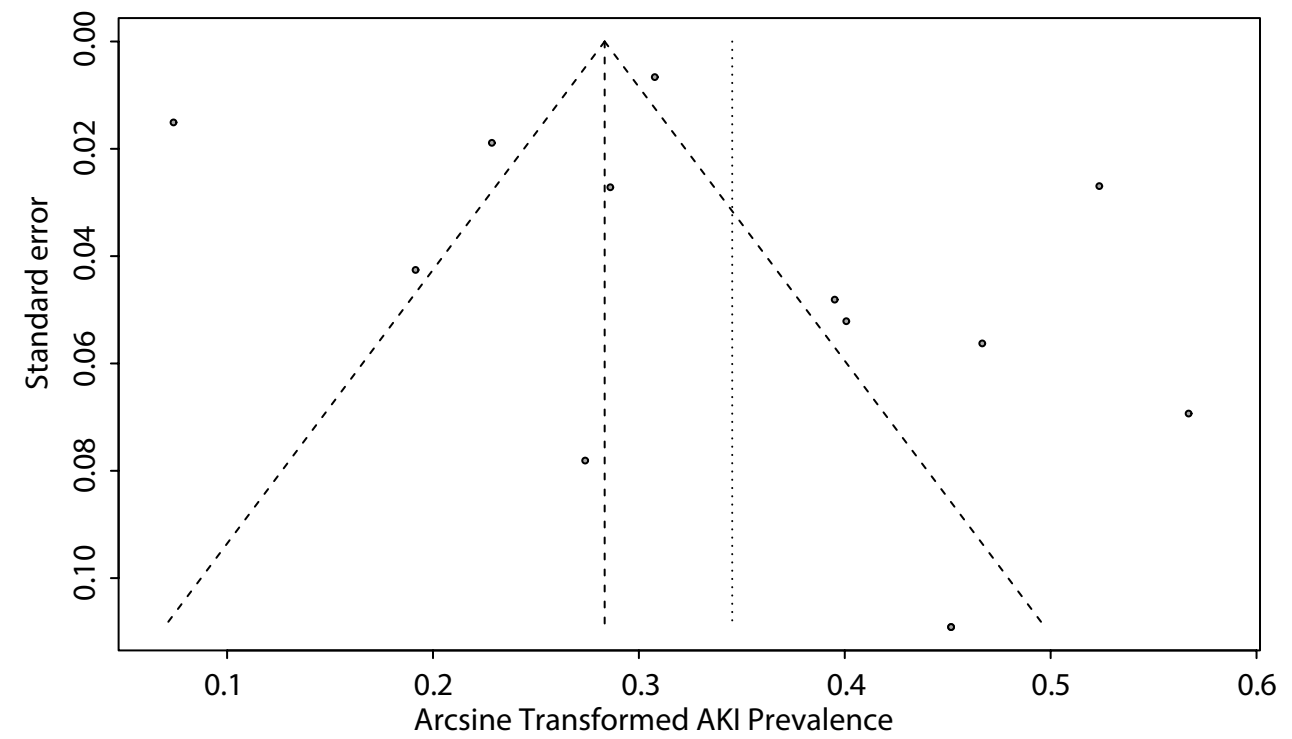

Supplement: Supplementary file 10 [file Data_Sheet_8.PDF]
